# Double Ovariotomy

**Published:** 1878-03

**Authors:** Samuel C. Plummer

**Affiliations:** Rock Island, Ill.


### DOUBLE OVARIOTOMY.

By Samuel C. Plummer, M. D., Rock Island, Ill.

*(Read at a Meeting of the Iowa and Illinois Central Disirict Medical Association, held at Davenport, Iowa, January* 10, 1878.)

On the 10th of October, 1877, I received the following letter from Dr. J. B. McLaughlin, of Delmar, Iowa:

"Dear Sir: My wife is suffering from what I think is an ovarian tumor; we are very anxious about her. Please come and see her at your earliest convenience. Let me know when you can come, and I will meet you at the station."

In compliance with this summons I visited the patient on the 13th of October, when I learned the following history of her case: Her age was 48 years; she had borne fourteen children at twelve confinements, having had two twin labors. Her health during this time had been good, with such exceptions as are common to child-bearing. Her last child was born in February, 1874, after which time menstruation had been irregular for twelve months, when it ceased entirely. After this, for six months she enjoyed the best of health. The menses then recurred, as was supposed at the time, in consequence of over-exertion. For a period of five or six months the flow would continue for from three to six days, and from seven to ten days would elapse during which time she was free from the discharge. About this time she began to suffer from cystitis, micturition was very painful, the pain in the bladder constant, and at times so acute as to be almost unbearable. In the month of January, 1877, the cystic trouble ceased and the menstrual flow became almost continuous, much of the time very profuse. In April an enlargement was perceptible in the right iliac region, which increased rapidly in size, displacing the abdominal viscera, and by the first of August it filled the entire right side of the abdomen, from the iliac to the hepatic region. It was nodulated; fluctuation in it could be detected, and its outline was easily traceable for more than two months before I saw the patient, and such was her condition when I first visited her on the 13th of October. I made a careful examination and found the abdomen greatly enlarged, too much so I thought to be exclusively caused by the tumor occupying the right side, and still there were no external signs of a second tumor, nor could I detect any by external manipulations. She however complained of greatest pain in the left iliac region; in fact there was little or no pain or tenderness on pressure on the right side. She was suffering from a very troublesome and exhaustive diarrhœa, which, with the uterine hemorrhage, was prostrating her very rapidly. She had no appetite; food she ate from compulsion, and she only slept when under the influence of large doses of soporific medicines. Such being her condition, I could find nothing to justify a hope that it could be bettered without operating for the removal of the tumor or tumors. I therefore advised that the operation be made as soon as possible.

Owing to the absence from home of three sons, a married daughter and a son-in-law, she determined not to have the operation performed till such time as they could all be with her.

Four days after (October 17th) I was notified that everything was in readiness. On the 18th, with Drs. Eyster and Carter as assistants, I proceeded to her home, and made the operation on the morning of the 19th.

Dr. Eyster administered chloroform, and when the patient was sufficiently anæsthetized, I made the usual incision along the linea alba, only large enough to ascertain to what extent, if any, adhesion resulting from peritoneal inflammation existed. The exploration developing no adhesion, I continued the incision up to within less than half an inch of the umbilicus, and down to within about the same distance of the symphysis pubis. This done, a second tumor came plainly into sight. At first, I was in doubt as to its character; its position was so nearly central, and its surface so smooth and glistening, that I was apprehensive it might be the uterus in a condition of fibroid enlargment. An exploration, however, soon satisfied me of its true character. It as well as the other tumor was incysted, but the cysts on its anterior surface were so pressed into contact with the abdominal walls as to give it a perfectly smooth and glistening appearance.

The next step in the operation was to dislodge the upper tumor, the one on the right side ; but it was so large that it could not be brought through the incision. I therefore resorted to paracentesis of the cysts to reduce their volume. In order to accomplish this, four of the largest cysts were emptied ; each contained from three to five pints of opalescent fluid mixed with pus. I used a long curved trocar and canula, and by this means prevented any part of the fluid from escaping into the peritoneal cavity. Having thus reduced its volume, the tumor was extricated. The pedicle was long, no doubt stretched by the upward pressure of the other tumor. Dr. Carter held this in position, and I proceeded to dislodge the other. I found it adherent over a large surface of its outer or left side, the adherent surface being as large as the palm of a man's hand. Having broken up these adhesions, I found that it also was too large for extraction through the incision, and in my effort to accomplish this, a large, and fortunately the anterior, cyst ruptured. It was so far raised, however, that its contents escaped over the abdomen of the patient, little, if any, finding its way back into the abdominal cavity. The contents of this cyst were entirely different from those of the other tumor, being composed of dark or coffee-colored gelatinous matter and pus. This sac also showed a high state of inflammatory action, and I have no doubt would have ruptured, and allowed its contents to escape into the abdominal cavity, within a very few days. Having completely sponged out this sac, I proceeded to drain others with the trocar and canula, until its volume was sufficiently reduced to allow it to be brought through the opening. I then used the "Atlee clamp," on the pedicle of the first or upper tumor, amputated and removed it. Then I passed a strong silk cord, double, through the pedicle of the other, tying it each way very tightly, and amputated above, sufficiently far to prevent slipping, and dropped it back into the abdominal cavity, bringing the ligatures out at the lower point of the incision, and safely fastening them on the abdomen and hip with adhesive strips.

Having passed a strong silk thread through the canula, the trocar was inserted and the instrument thus armed was carefully carried down into the cul-de-sac of Douglas, directed by the hand. With the index finger of the right in the vagina, the thinnest part of the partition was found, when an assistant forced the instrument through the vaginal wall. The thread was caught in the vagina and, with the canula held in position, the trocar was withdrawn. The drainage tube tied to the abdominal end of this thread was then drawn through the canula, through the vagina, and when the vaginal end protruded from the vulva an inch or more, it was fastened to the thigh by ligature and adhesive strips. The canula was now withdrawn and the abdominal end of the drainage tube was coiled and dropped into the cul-de-sac of Douglas.

The drainage tube used was made of very fine, soft india rubber, somewhat less than 3-16 of an inch in diameter, the caliber being about 1-16 of an inch and fifteen to eighteen inches in length. Perforations were made into this tube with spaces of one to one and a half inches between each, its entire length. The drainage was perfect and very profuse. The tube was accidently dislodged on the third day after the operation but the drainage continued through the puncture, gradually deminishing, until it entirely ceased in about four weeks from the time the operation was made.

The operation being completed, thus far, the edges of the abdominal wound were brought together and held by interrupted sutures, and long bands of india rubber adhesive plaster. Lint wrung out of hot water was applied over the abdomen as warm as could be borne and over this was spread a piece of oiled silk large enough to cover the entire abdomen.

The temperature of the room was 70° throughout the time of the operation, notwithstanding which the patient was chilly when it was completed. I therefore ordered bottles filled with hot water put into the bed about her feet and legs, and had additional bed clothing spread over her. She soon rallied from the effects of the chloroform and when we bade her good-bye at fifteen minutes before eleven o'clock in the forenoon, she expressed herself as feeling very comfortable.

The solid matter, with a portion of the contents of the tumors, weighed eighteen pounds. I believe they, with their entire contents, would have weighed at least twenty-five pounds. Before leaving her I administered one-third of a grain of sulphate of morphia in solution by hypodermic injection; this was done in anticipation of the pain to be expected when reaction was established. I also directed dressings of lint, wrung out of warm carbolized water, to be applied, and continued over the abdomen; the oiled silk to be kept on over this dressing, and the temperature of the room to be kept as near as possible at 70°. All these directions were faithfully carried out by the husband of the patient, Dr. McLaughlin, until she was so far recovered as not to require their strict observance.

I was apprehensive for the welfare of my patient because of the severe and extensive peritoneal inflammation found present on opening into the abdominal cavity. The peritoneal covering of the intestines and of the omentum, that lining the abdomen on the left side and that covering the tumor of the left side, was in a state of highly acute inflammation. This inflammation was so extensive and acute that Drs. Eyster and Carter agreed with me in considering the chances for recovery as being against her.

When Dr. Eyster commenced administering the cloroform, Dr. Carter was watching and timing her pulse, which was one hundred and sixty per minute; small and irregular. It improved under the influence of the chloroform, and when she had rallied from its effects, after the operation was completed, it was one hundred and twenty, more regular, and its volume much improved.

Her condition was reported to me from time to time as follows:

"October 20th, 5½ o'clock a. m.—Your patient has recovered nicely from the effects of the chloroform; vomited some during the night; was thirsty and drank, we think, too much water; is thirsty yet, but has not vomited in the last two hours; she felt better and easier during the night than she has for many days prior to the operation, and is now free from pain. We gave hypodermic injection of solution of morphine at 2 o'clock this morning. Indications all favorable so far, and we hope they may continue so. There is profuse discharge from the vagina. Yours truly, GEORGE E. MYERS."

Mr. Myers, a son-in-law of the patient, is a student of medicine, and with his wife, took care of the patient during the first night after the operation.

"Oct. 21st, Sunday morning.—Patient was very restless during yesterday; at 6 o'clock p. m, her pulse was 140 per minute, very weak and irregular. She vomited some during the day; had hiccough; was very thirsty; had clammy perspiration; was very restless, and it required a larger quantity of morphine than usual to produce quiet. She had good refreshing sleep during the night; pulse 112 this morning; the hiccough and vomiting have nearly ceased. She has taken some nourishment and feels better.

"Oct. 22d, Monday morning.—She rested well during last night. Her pulse at 6 o'clock last evening was 100 per minute; is 100 this morning, soft and regular. She thinks she must have something to eat. The drainage during yesterday and last night was very profuse. The drainage tube came away last night; it is not known what brought it away; she thinks the violent efforts in vomiting dislodged it. The drainage continues through the puncture. The wound of the abdomen looks well. She urinates often; her bowels have been moved this morning, the first since the operation. Yours, etc., J. B. McLaughlin."

"Oct. 23d, 6 o'clock a. m.—Patient is feeling as well as could be expected; pulse 100 per minute, regular, full and soft; appetite improving, vomiting and hiccough have entirely ceased. Skin moist, urinates frequently; bowels have not moved since yesterday morning; complains of some pain where the tumors were and at the sutures; a little pus discharging at the sutures. The drainage continues, but diminished in quantity, and is higher colored.

"Oct. 24th, morning.—We are greatly encouraged. Patient ate all she was given yesterday and relished her food; has very little pain this morning; discharge from drain is gradually diminishing; urinates freely; bowels have not been moved; slight flatulency; pulse 100, full and regular; symptoms all good.

"Oct. 25th, Thursday morning.—Pulse 94. She did not rest so well last night; complains of pain in the incision and in left side; urine has a strong ammoniacal odor; bowels have not been moved. I will administer an enema to-day; her strength is gaining, has appetite enough.

"Oct. 27th, morning. Her bowels were well moved on Thursday evening. I feel very much encouraged this morning; drainage continues; pus discharging from incision and from the clamped pedicle; urine not so strongly ammoniacal; pulse 96 last night and the same this morning, soft and regular."

This is the last report I received from the doctor as he met with an accident that will be explained in the following reports, written by his daughter. The accident resulted in the loss by amputation of the middle finger of his right hand.

"November 1st.—Mother is doing well. The clamp came off on Sunday morning (October 28th, nine days after the operation was performed). While cleansing the clamp, father pricked the middle finger of his right hand with one of the pins. During Sunday night, he had frequent chills; has suffered pain all the time since. We think he is in greater danger than mother, and want you to come and see him as soon as possible." I went in compliance with this summons on the afternoon of November 1st, and found the doctor in great pain; the hand and forearm much inflamed and swollen, however, not so seriously but that I hoped the finger could be saved. Mrs. M. was feeling so well that she was anxious to leave her bed and sit up in a chair.

November 2nd.—I again visited the Doctor and his wife, and gangrene having set in, amputated the finger at the metacarpo-phalangeal joint. Found Mrs. M. doing well and feeling well.

"November 7th.—Mother is still improving; the ligature came away yesterday morning (Nov. 6th, eighteen days after the operation); the wound is nearly closed, discharging very little pus. Father's hand is doing well."

I received letters from time to time from the daughter, informing me as to her mother's condition, which was as satisfactory as could be desired up to the 5th of December, when I was summoned to see her on account of cystic trouble. On my arriving at her home, I learned she had been suffering greatly for about a week; only was able to bear the pain when under the influence of large doses of morphine. Micturition was attended with intense pain, and she was unable to retain the urine more than ten to fifteen minutes; the quantity voided amounted to from two to four or five drachms each time.

The urine on examination with the urinometer showed a specific gravity of 20°; litmus paper showed an excess of acid. Epithelial débris could be seen floating upon its surface; tests by acid and boiling showed the presence of albumen and but little if any sugar. She was without appetite for food and had nausea, tongue heavily furred, abdomen swollen and tympanitic, bowels constipated, the liver and spleen enlarged and painful on pressure. The wound of the abdomen was entirely healed, and there was no tenderness over the site of the ovaries removed, but great pain on pressure over the bladder. The drainage through the puncture by the vagina had entirely ceased. I advised powders of opium and calomel, one to be given every two hours, and a dose of castor oil ten hours after taking the last of the powders.

I recommended also the use of f. ext. of pareira brava, or an emulsion of balsam of copaiba, after the bowels had been well moved by the cathartic. Also injections of tepid water with morphine into the bladder.

"December 10th.—Mother is quite comfortable this morning. She passed gravel last night."

This calculus was about an inch in length, and three-eighths of an inch in its greatest diameter, tapering from its middle to its extremities, both of which were rounded; its surface was rough, grayish in color, fragile and soluble in muriatic acid.

To the use of the drainage tube I credit the recovery. It perfectly performed its purpose, and without it I am fully persuaded success would have been impossible.

The quantity of sero-sanguineous matter discharged through the tube while *in situ*, and after its dislodgment through the puncture, was simply enormous for the first seven or eight days. The quantity was too great to have been taken up by absorbent action, and this great quantity too, escaped after every means had been used to prevent the escape of fluids into the abdominal cavity, and after all that could be removed with the long, pointed, hard rubber syringe, used for such purposes, had been evacuated.

After this excessive discharge had ceased, the drain continued for some three weeks, diminishing each day until it entirely ceased, and the puncture closed.

In every instance where death has resulted from ovariotomy, so far as my experience goes, and so far as I have been able to learn from others, septicæmia has been the cause. Therefore, to prevent death from that cause, the drainage tube should be used. It should be used in every case, no matter how favorable and how promising it may be, for without it no patient is free from that danger, and with it that danger is almost, if not entirely, avoided.

I consider it, to say the least, hazardous practice to ligate the pedicle or pedicles, cutting the ligatures short, and dropping them with the stumps back into the abdominal cavity; for in very many, if not in all cases, the ligatures thus left are the cause of much irritation, if not, as is generally the case, of peritoneal inflammation, with its consequences. When recovery does follow such practice, it is slow, often not until the ligature has sloughed its way through the vaginal or intestinal walls.

In this case, I attribute the recovery, first, to the perfect drainage of all matters from the peritoneal sac that could, by remaining there, have resulted in septicæmia; and, second, to the withdrawal, after separation, of the ligature used on the stump dropped into the abdominal cavity.

